# Menstrual cycle hormones and oral contraceptives: a multimethod systems physiology-based review of their impact on key aspects of female physiology

**DOI:** 10.1152/japplphysiol.00346.2023

**Published:** 2023-10-12

**Authors:** Alysha C. D’Souza, Mai Wageh, Jennifer S. Williams, Lauren M. Colenso-Semple, Devin G. McCarthy, Alannah K. A. McKay, Kirsty J. Elliott-Sale, Louise M. Burke, Gianni Parise, Maureen J. MacDonald, Mark A. Tarnopolsky, Stuart M. Phillips

**Affiliations:** ^1^Department of Kinesiology, McMaster University, Hamilton, Ontario, Canada; ^2^Mary MacKillop Institute for Health Research, Australian Catholic University, Melbourne, Victoria, Australia; ^3^Institute of Sport, Manchester Metropolitan University, Manchester, United Kingdom; ^4^Department of Pediatrics, McMaster University Medical Center, Hamilton, Ontario, Canada

**Keywords:** endocrinology, exercise, female, human, menstrual cycle

## Abstract

Hormonal changes around ovulation divide the menstrual cycle (MC) into the follicular and luteal phases. In addition, oral contraceptives (OCs) have active (higher hormone) and placebo phases. Although there are some MC-based effects on various physiological outcomes, we found these differences relatively subtle and difficult to attribute to specific hormones, as estrogen and progesterone fluctuate rather than operating in a complete on/off pattern as observed in cellular or preclinical models often used to substantiate human data. A broad review reveals that the differences between the follicular and luteal phases and between OC active and placebo phases are not associated with marked differences in exercise performance and appear unlikely to influence muscular hypertrophy in response to resistance exercise training. A systematic review and meta-analysis of substrate oxidation between MC phases revealed no difference between phases in the relative carbohydrate and fat oxidation at rest and during acute aerobic exercise. Vascular differences between MC phases are also relatively small or nonexistent. Although OCs can vary in composition and androgenicity, we acknowledge that much more work remains to be done in this area; however, based on what little evidence is currently available, we do not find compelling support for the notion that OC use significantly influences exercise performance, substrate oxidation, or hypertrophy. It is important to note that the study of females requires better methodological control in many areas. Previous studies lacking such rigor have contributed to premature or incorrect conclusions regarding the effects of the MC and systemic hormones on outcomes. While we acknowledge that the evidence in certain research areas is limited, the consensus view is that the impact of the MC and OC use on various aspects of physiology is small or nonexistent.

## Graphical abstract

**Figure:**
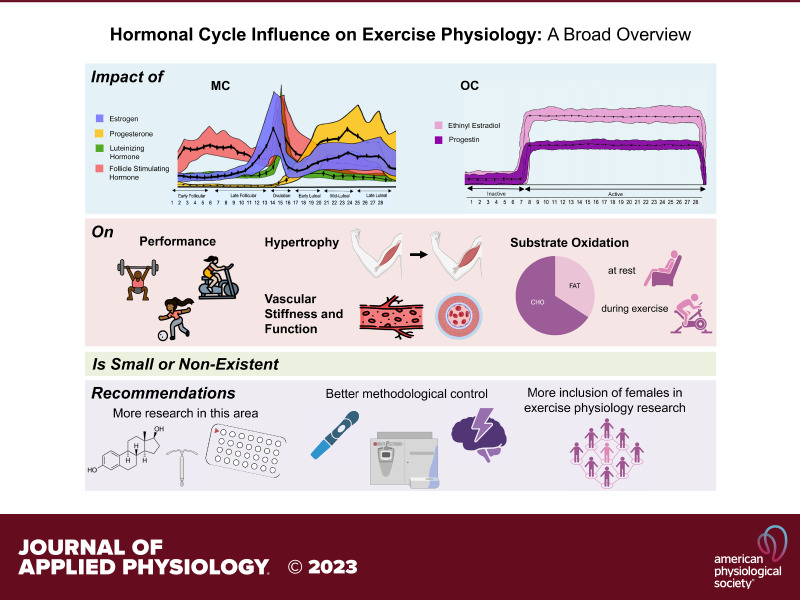

## INTRODUCTION

Females are historically underrepresented in exercise physiology research compared with males (1–5); the basis for this sex-based exclusionary bias is uncertain (1–5). We propose that this disparity may be partly due to the hypothesis that menstrual cycle (MC)-based hormones influence exercise metabolism, muscular hypertrophy, and/or vascular responses to an extent substantial enough that it renders the inclusion of females in exercise physiology research “cumbersome.” Considering the MC requires understanding the MC phase, studying males is “less work.” Some have argued that control for MC phase may be necessary in some cases (5)—the main questions addressed in this review concern aspects of female-based exercise responses across MC phases. Topics include substrate oxidation, exercise performance, muscle physiology in response to resistance training, and vascular physiology.

If MC phase-specific differences exist, a key question arises: are these changes substantial enough to require the inclusion of MC phase in an experimental design, as they affect, beyond measurement error, the outcome? If this is the case, then it would require testing women, at a minimum, in two separate phases of their MC to ascertain a follicular phase- and a luteal phase-specific measurement. However, whether that is the case is uncertain.

To date, no systematic reviews objectively have evaluated the state of the research on MC phase and physiological responses to exercise. In an area with robust evidence and available data, we have undertaken a meta-analysis to examine substrate oxidation responses across different phases of the MC, both at rest and during exercise. In areas with less evidence, we offer narrative insights into the effect of MC phase on exercise performance, muscle physiology, the cardiovascular system, and arterial responses. Furthermore, we address how oral contraceptive (OC) use might impact metabolism in specific areas. OC users have been studied in the active and inactive (placebo) phases, and differences have been proposed to be evident. However, we note that in the area of OC research, there is scant material on effects, particularly on OC of different generations, which may affect aspects of female physiology; nonetheless, we attempt to synthesize the current body of knowledge in this area. Due to the complex hormonal environments that characterize the MC, we rely almost exclusively on human female-derived data to form our conclusions in this review.

**Figure 1.**
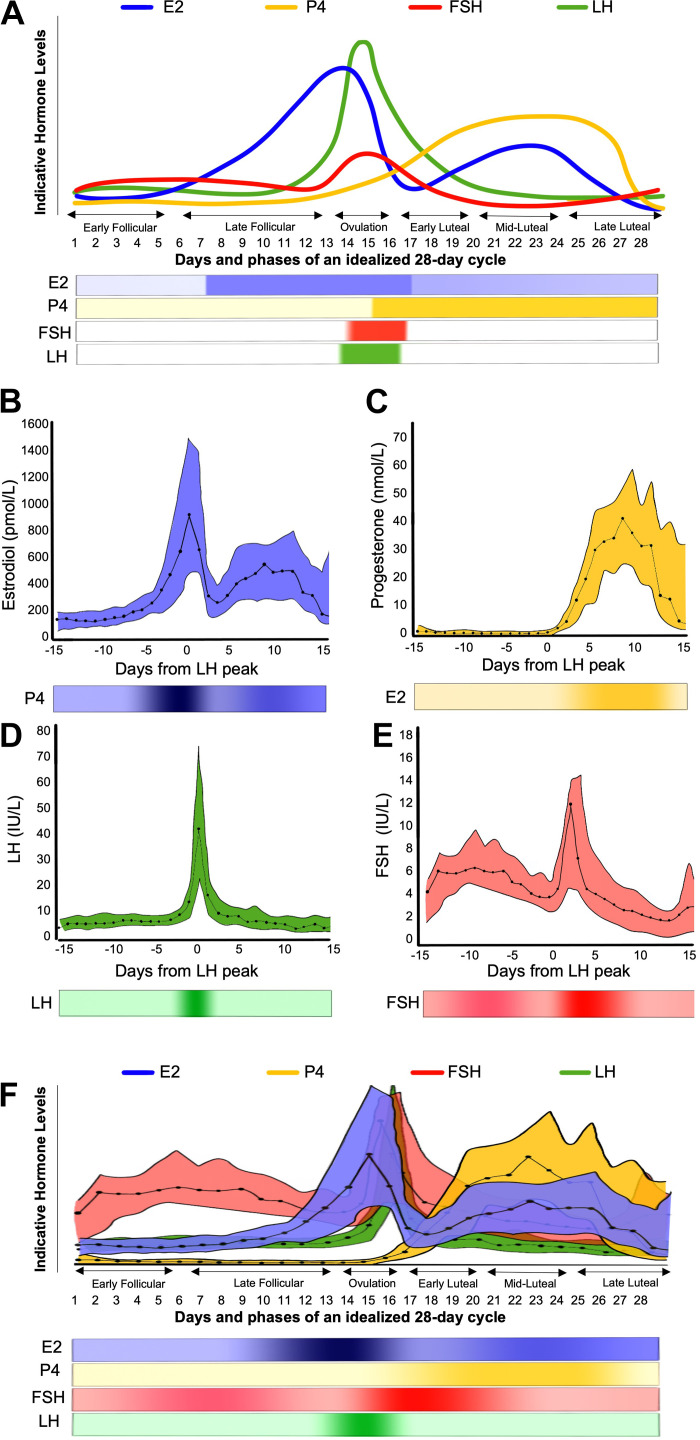
A schematic of dominant sex hormone concentrations across the MC in naturally cycling women. *A*: hormonal fluctuations across an idealized (textbook) 28-day MC, with ovulation occurring on day 14. Hormone values in daily serum samples across the MC for naturally cycling women, adapted from Stricker et al. (6). Estradiol (*B*); progesterone (*C*); luteinizing hormone (*D*); follicle-stimulating hormone (*E*); and estradiol, progesterone, luteinizing hormone, and follicle-stimulating hormone concentrations overlapped (*F*). Solid lines represent median values; the colored area highlights the 95% confidence interval range. All figures are accompanied by a visual color gradient depicting changes in the respective hormone concentrations across the MC. E2, estradiol; FSH, follicle-stimulating hormone; LH, luteinizing hormone; MC, menstrual cycle; P4, progesterone. (6–8).

The MC is characterized by regular fluctuations in sex steroid hormones, namely, 17β-estradiol (E2), progesterone (P4), follicle-stimulating hormone (FSH), and luteinizing hormone (LH). It comprises two phases, the follicular and luteal phases, demarked by the onset of ovulation. These phases can be divided into subphases: early follicular, late follicular, ovulation, early luteal, midluteal, and late luteal (Fig. 1*A*). Although a relatively large body of physiology research has examined MC phase and how it affects various aspects of muscular and vascular metabolism, an underappreciated issue lies in acknowledging the substantial variability between (and within) females regarding MC length, ovulatory events, and relative hormonal concentrations (9–11). Although the “textbook-level” teaching of MC endocrinology (Fig. 1*A*) highlights stylized average responses, measured profiles of E2, P4, LH, and FSH across the MC exhibit a wide distribution (Fig. 1, *B*–*E*). These data, which include median and 95% confidence intervals, illustrate the complexity of isolating specific physiological effects to distinct phases of the MC and ascribing those effects to a particular hormone (11, 12). Figure 1*F* shows the combination of the hormonal profiles from Fig. 1, *B*–*E*. Figure 1*F* highlights a degree of overlap, showing the complexity of defining a phase by a certain hormonal environment and subsequently attributing phase-specific physiological effects to a singular “dominant” hormone. In this review, we rely almost exclusively on human female-derived data to form our conclusions. While we acknowledge that rodent- and/or cell-based models can offer unprecedented insight into mechanisms of specific hormones’ mechanisms in isolation or combination, the relevance to humans is sometimes unclear. These models often depict the complete absence or presence of a particular sex hormone in influencing specific molecular processes. The caveat in using nonhuman models is that hormonal states of presence (augmentation via pumps to achieve supraphysiological levels) or a complete absence of hormones (ovariectomy) do not, in our view, readily translate to the normal MC or hormonal mechanisms in females. In this review, we rely, to the extent possible, on human-derived data to form our conclusions.

## WHAT IS A NORMAL MC?

Before discussing specific aspects of female physiology, it is prudent to establish what constitutes a normal menstrual cycle. The etymology of eumenorrhea is Greek: eu meaning “good,” meno meaning “month,” and rrhea meaning “flow” (i.e., good monthly flow). Colloquially, eumenorrhea is described as normal or regular menstruation, whereas menorrhea means normal menstrual flow in a medical setting. Nonetheless, what constitutes a normal/regular MC is seldom defined.

**Figure 2.**
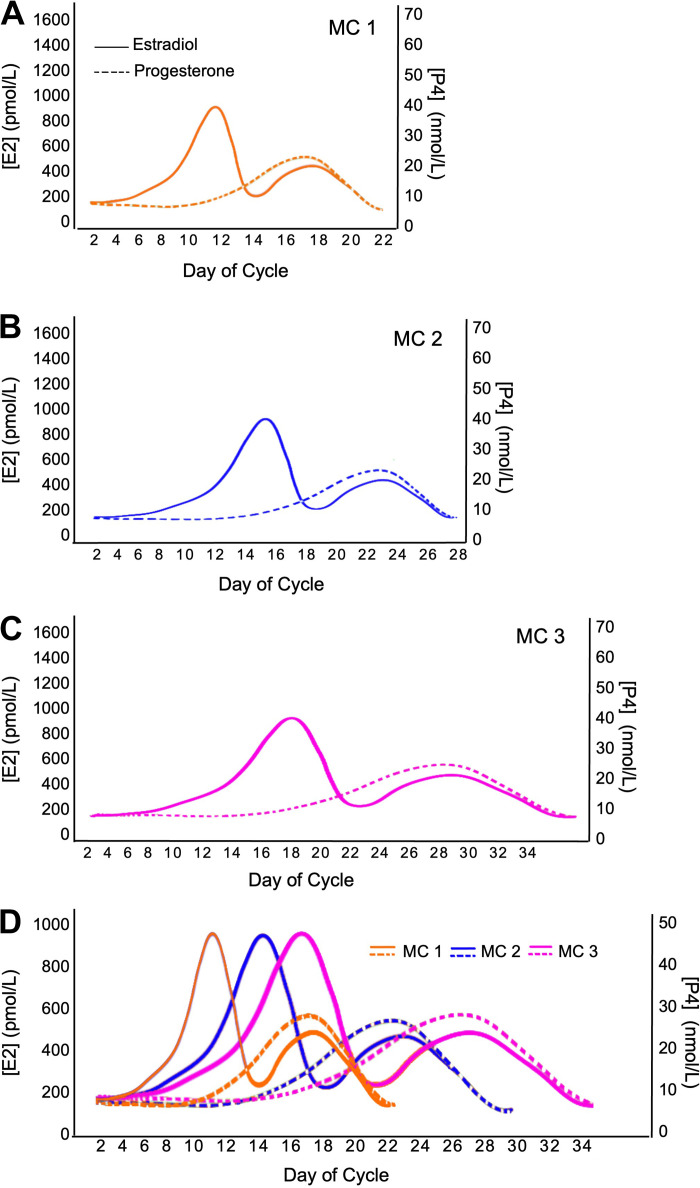
A schematic depiction highlighting the potential variability in MC length in three females. Normal MC length can range from 21–35 days (10, 13). A normal MC that is: 21 days (*A*); 28 days (*B*); and 35 days in length (*C*). *D*: MCs of varying length (*A–C*) plotted on a single graph to highlight the variability across MCs, showcasing variations within and between individuals. E2, estradiol; MC, menstrual cycle; P4, progesterone.

**Figure 3.**
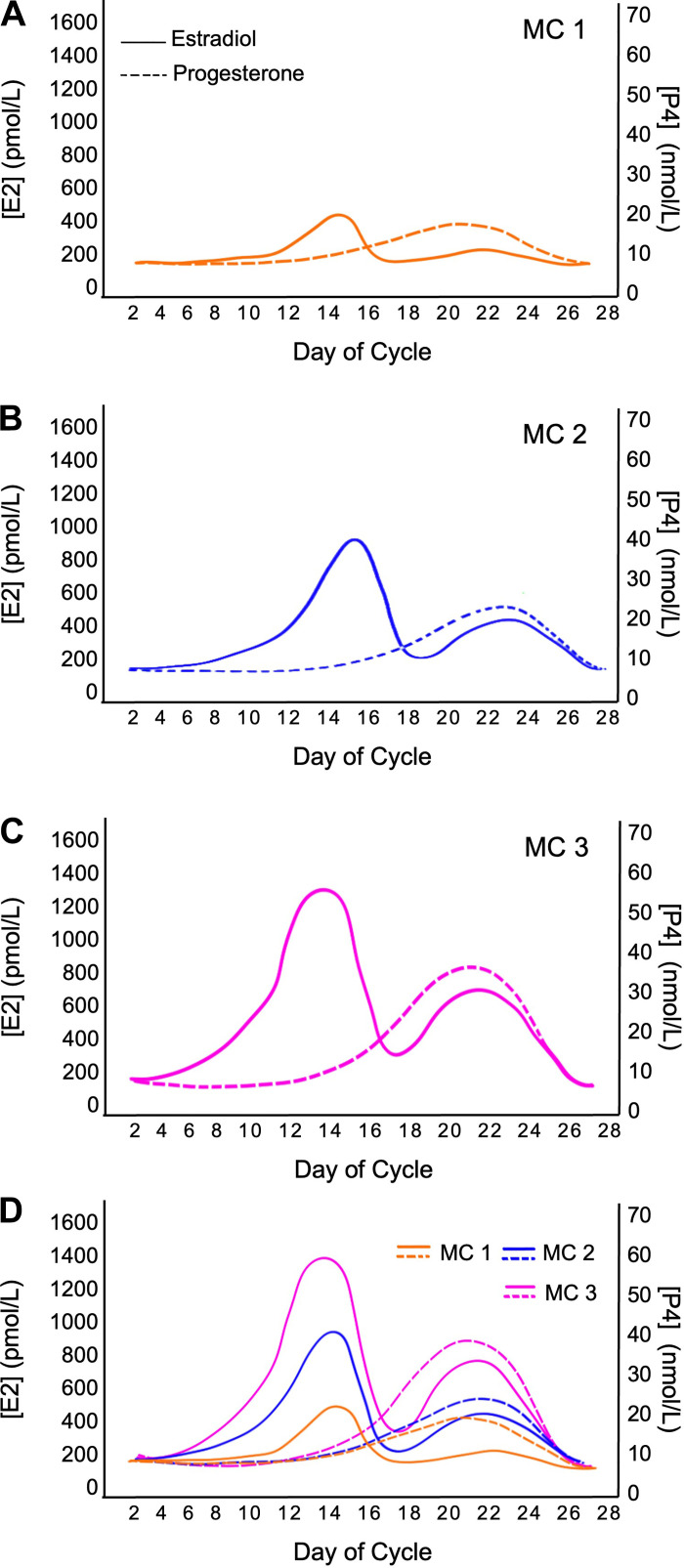
A schematic showing the potential variability in hormonal concentrations between MC. Drawn using data from Ref. 6. A normal MC with lower (*A*), average (*B*), and higher (*C*) levels of estrogen and progesterone throughout the MC. *D*: MCs with varying hormone concentrations (MC 1–3) plotted on a single graph to highlight the variability in hormone levels across MCs, showcasing variations within and between individuals. E2, estradiol; MC, menstrual cycle; P4, progesterone.

From a research perspective, eumenorrhea refers to female participants with a MC that satisfies the following criteria: MC lengths ≥21 days and ≤35 days, evidence of luteinizing hormone surge, correct hormonal profile, and no hormonal contraceptive use for 3 mo before recruitment (10). Despite adhering to the criteria as mentioned above, substantial variations in cycle characteristics of eumenorrheic women can exist. Figures 2 and 3 illustrate the possible cycle lengths and hormonal patterns for eumenorrheic participants in the same study. When examining Fig. 2, what becomes abundantly clear is that cycle length can vary up to 14 days, both between individuals and, albeit less usually so, within the same individual. Furthermore, it is important to reiterate that even in individuals with a consistent MC length, the relative changes in E2 and P4 concentration could vary greatly (Fig. 3). This point is important to note as it may substantially impact tissue-level postreceptor mechanisms hypothesized to be important in affecting metabolism (14–16). Thus, even if we were to measure systemic hormone concentrations and infer biological effects, we also have less data on how systemic hormonal concentrations are related to local tissue-level concentrations. It may be that tissue-level concentrations and postreceptor signaling would be more relevant to physiological events (14–16) than blood levels.

Independent of blood measures, establishing eumenorrhea in research settings requires both proactive and reactive strategies. In the former case, researchers must be mindful of completing all necessary steps to establish consistent eumenorrheic status. For example, this process could be aided with cycle-tracking applications or ovulation testing. Meanwhile, participants must alert the investigator to an unanticipated menstrual event and make themselves available for testing on short notice. When testing cannot be scheduled in the required phase, the study must be extended by another cycle, thus adding to the burden of participation (10).

In addition to the difficulties in establishing eumenorrhea and the variation in cycle characteristics even when eumenorrhea has been established, researchers also need to consider other methodological factors associated with recruiting and testing eumenorrheic participants (10). Importantly, double-blinding to the MC phase of testing is not possible, but posttesting blinding of the researcher during analysis is possible. Ovarian sex hormone concentration can be influenced by prior activity, dietary energy restriction, smoking, and stress, which would ideally be controlled or standardized to the degree possible (10). Despite using the terms eumenorrhea and MC phases, the majority of previously published studies have recruited and tested “naturally menstruating” women (i.e., women who experience menstruation with MC lengths ≥21 days and ≤35 days, but without confirming any or many of the remaining criteria for eumenorrhea) and have only been monitored to verify their MC using one phase (10). The result is that there is only an estimate of the timing and assumed occurrence of all other MC phases, which could vary substantially (Figs. 1, 2, and 3). These assumptions have profoundly affected the quality of the research evidence to date (9, 10) and have, in our view, led to potentially spurious conclusions around the influence of E2 and P4 on various outcomes. Thus, given the intra-and interindividual variability across the MC in eumenorrheic women, adequate methods for testing and tracking MC phase are needed when researching this topic. For further information on the standard of practice methods in MC tracking, please see the following review (10).

## SUBSTRATE OXIDATION ACROSS THE MC

Substrate oxidation during exercise differs between sexes, with females generally being proposed to derive more of their exercising energy from lipids, as evidenced by a lower respiratory exchange ratio (RER) (17). In addition, females exhibit a higher glucose rate of disappearance [Rd; (18)] and higher intramyocellular lipid oxidation (19, 20). These differences are often attributed to circulating estrogen concentrations, particularly during variations across the MC (21).

Estradiol alone promotes increased fat and decreased carbohydrate oxidation at rest and during endurance exercise (22, 23). Conversely, P4 may be antagonistic to the effect of E2, promoting increased carbohydrate oxidation (24). As a result of these findings, the ratio of E2 to P4 has been implicated as an important factor in moderating MC effects on substrate metabolism. For example, a study looking at differences in RER during moderate-intensity cycling showed no significant difference between phases, presumably as a result of similar ratios between E2:P4 found between phases in this population (57).

Research investigating substrate oxidation while taking OC in different phases, active versus placebo, is sparse and has primarily studied comparisons of participants using OC versus nonusers. Certain OC formulations may have multifaceted effects on metabolism. For instance, they may promote lipolysis and decrease peripheral glucose uptake and utilization (56, 90), lower blood glucose concentrations, increase systemic free fatty-acid concentrations, and decrease glucose rate of appearance.

Furthermore, OC use may increase cortisol concentrations during exercise, a factor known to enhance lipolysis and reduce peripheral glucose uptake and utilization during exercise; this suggests a carbohydrate-sparing effect of OC use (55). Other investigations report no phase-related differences in substrate oxidation in OC users (55). These inconsistencies may be a result of the type of OC formulation used (monophasic and triphasic), the generation (1st–4th) of OC, the dose of exogenous hormone, and/or the duration of OC use within an individual (66). Much like the MC, the ratio of synthetic estrogens to progestins may also be an important consideration in selecting fuels with OC use.

Given the robust amount of data comparing substrate use across the MC and with/without OC use, we elected to conduct a systematic review and meta-analysis to determine whether MC phase affected substrate oxidation. Details of the methods used in this meta-analysis can be found in Supplemental Figs. S1 and S2 (all Supplemental material is available at doi.org/10.6084/m9.figshare.23279927). In brief, the systematic review and meta-analysis were conducted following the preferred Reporting Items for Systematic Review and Meta-Analysis (PRISMA) guidelines and was registered on the International Prospective Register of Systematic Reviews (PROSPERO) under registration #CRD42021237018. The search strategy was determined in collaboration with a university librarian. The article selection and screening results are detailed in Supplemental Fig. S1. Articles were obtained and imported into Covidence (Melbourne, Australia) for the title and abstract review, followed by a full-text review.

The population included in this investigation were female participants aged 18 or older, with no restriction as to health status (i.e., healthy and clinical populations included). Only premenopausal female participants who were naturally cycling or using cyclic hormonal contraceptives were included, with at least two phases of the hormonal cycle examined.

Observational research trials that assessed the following outcome measures at rest or during moderate-intensity continuous exercise were included. Within-subject study designs were also included, where participants exercised during at least two phases of the hormonal cycle. Resting data were included if directly measured in individuals sitting or lying down for a minimum of 5 min. Resting data were excluded if obtained acutely after exercise or fasting duration was >16 h. Moderate-intensity continuous exercise was defined as occurring for at least five consecutive minutes into a constant-load exercise bout, below an intensity of ventilatory or lactate threshold, or 70% V̇o_2peak_ (25, 26). Data were excluded if substrate metabolism was experimentally manipulated, collected before 5 min into exercise, if a respiratory steady state could not be achieved during exercise, or if incremental exercise tests or exercises involving >1 modality (e.g., circuits) were used. Studies where substrate metabolism was manipulated (e.g., nutritionally or through intravenous infusions) were excluded, but placebo or control data were included.

Substrate oxidation across at least two hormonal subphases was compared. The most commonly studied phases included the early follicular phase (∼days 1–7 of the menstrual cycle) when endogenous estradiol and progesterone are low, the late follicular phase/ovulation phase (∼days 14–16 of the menstrual cycle) when endogenous estradiol is high but progesterone low, and the midluteal phase (∼7 days postovulation) when both estradiol and progesterone levels are high. Similarly, OC pill cycles are often studied by examining the low-hormone phase when exogenous estrogen and progesterone are low (placebo/hormone withdrawal phase) and when hormones are both high (active phase). The primary outcome was substrate oxidation during moderate-intensity continuous exercise, and the secondary outcome was substrate oxidation at rest. Substrate oxidation was assessed via nonprotein RER determined using indirect calorimetry and specific substrate oxidation, such as carbohydrate (glucose) and fat, where available. The rate of disappearance of labeled glucose was accepted as its oxidation rate during exercise (18). The net breakdown (i.e., pre- and postexercise) of glycogen and triglycerides was also considered to be oxidized during exercise (28, 56).

From 2,845 articles, after removing duplicates and after the title and abstract screening, 707 studies remained. Of these, 117 full-text articles were assessed for eligibility, 62 of which were excluded due to missing outcome measures, duplicate studies, no comparison across MC or OC phase, incorrect intervention, including the abstract only, or presented as a thesis. A total of 55 studies with 928 participants met the inclusion criteria and were included in the review (see Supplemental Fig. S1). Of the 55 studies included in the review, 25 reported the relation of the menstrual phase with substrate oxidation at rest and 25 during exercise (*n* = 487 participants for rest, *n* = 483 participants for exercise). Six studies reported the relation of the OC phase with substrate oxidation during exercise (*n* = 51). There were insufficient studies investigating substrate oxidation across the OC phase during rest; thus, these results are not depicted here, and the overall trend of the data is presented for information (but would be of low quality). The quality of the evidence from the studies included in this review was classified as “moderate” in quality (0% “very low”; 28% “low”; 70% “moderate”; 2% “high”).

Visual inspection of the funnel plots did not identify any substantial asymmetry in MC phase effects on substrate oxidation at rest, during moderate-intensity continuous exercise, or OC phase effects on substrate oxidation during exercise (Supplemental Fig. S2).

Data were presented as mean ± standard deviation (SD). Studies that reported data as mean ± standard error of the mean (SE) were converted through the following equation: SD = SE × sqrt(N) where N = sample size within that study. Studies that included multiple outcomes were included in the same analysis, with the sample size proportionally decreased (i.e., if a study included two outcomes, the sample size would be halved to include both outcomes in the analysis). Pairwise meta-analyses examined the effect of MC phase (follicular vs. luteal) and OC phase (inactive vs. active) on the standardized mean difference (SMD) of each substrate oxidation measurement. The SMD was selected over the mean difference to allow for standardization across multiple methods for substrate oxidation. The SMD was weighted using the inverse variance method and pooled using a random-effects model. Participants in the follicular phase (for MC analyses) or active phase (for OC analyses) were used as the comparator, such that a positive SMD corresponded with increased carbohydrate oxidation in luteal or inactive phase participants. Significance was set to *P* < 0.05 with effect sizes examined according to Cohen guidelines (SMD of 0.2, 0.5, and 0.8 correspond to small, medium, and large effect sizes, respectively). Subgroups were determined a priori and were analyzed when heterogeneity was high (I^2^ ≥ 50%), including nutritional status [fasted vs. postabsorptive (2–4 h) vs. fed], training status (sedentary vs. recreationally active vs. trained), exercise duration (5–10 min vs. 30 min vs. 60 min vs. 90 min), exercise intensity (30–35%, 40–45%, 60–65%, and 70–75% V̇o_2peak_), and comparisons across methodologies (e.g., whole body vs. skeletal muscle measures).

Study quality was assessed using a modified Downs and Black (29) checklist. Two investigators independently assessed study quality using the Downs and Black checklist, and any discrepancies were solved by a third reviewer. The study quality for each outcome was assessed using the following scale: “high,” “moderate,” “low,” or “very low” quality. Certainty of the evidence was determined using the Grading of Recommendations Assessment, Development, and Evaluation (GRADE) criteria using the GRADEPro tool. As per GRADE criteria, certainty of evidence was downgraded if: the risk of bias was serious; if 50% of the weight of the pooled SMD contributing to the meta-analysis was “low” or “very low” quality from the Downs and Black checklist (29); if inconsistency of the results was serious when heterogeneity assessed (I^2^ ≥ 50%); if there was indirectness of results; if imprecision was “serious” if the analysis’ 95% CI crossed the line of no effect; and if publication bias was detected using visual analysis of funnel plots. Studies were upgraded if there was *1*) a large magnitude of effect (SMD = 0.8 or higher), *2*) a dose-response, or *3*) confounding factors would reduce the effect; criteria (2) and (3) could only be used to upgrade a study if the study was not previously downgraded. All observational studies started with “low” quality as a nonrandomized control trial and downgraded/upgraded accordingly.

**Figure 4.**
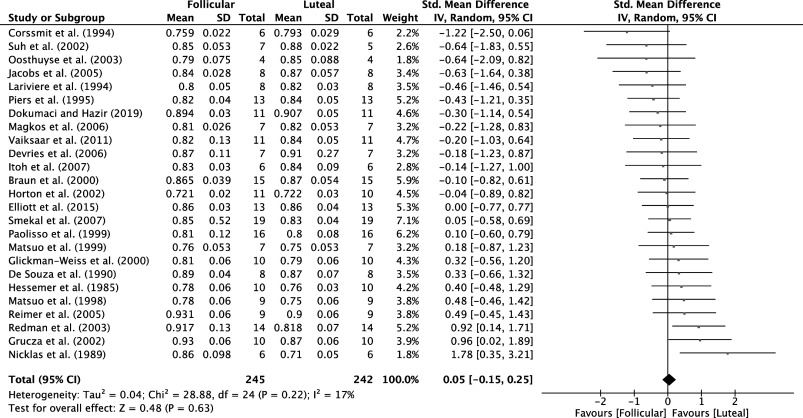
Forest plot of meta-analysis comparing RER at rest in the follicular vs. luteal MC phases; data extracted from Refs. 28, 30–53. MC, menstrual cycle; RER, respiratory exchange ratio.

**Figure 5.**
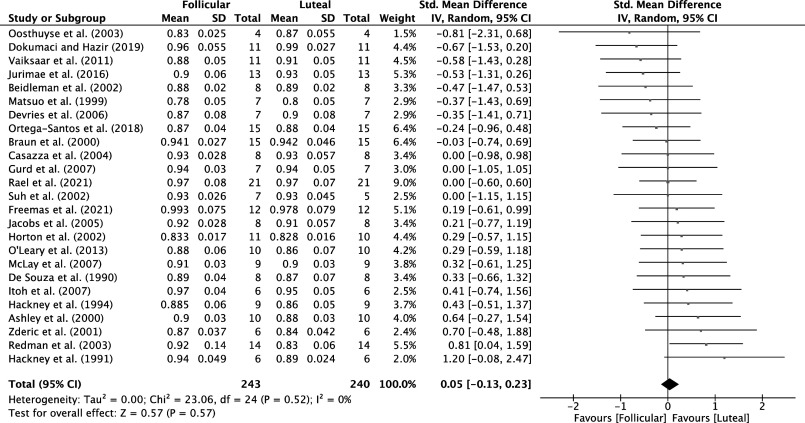
Forest plot of meta-analysis comparing RER during moderate-intensity continuous exercise in the follicular vs. luteal MC phases; data extracted from Refs. 22, 24, 30, 32, 33, 37, 38, 42, 44, 47, 50–64. MC, menstrual cycle; RER, respiratory exchange ratio.

Analysis of MC phases from 25 studies for trials with participants at rest (*n* = 487 participants) and 25 studies for MC trials with participants during exercise (*n* = 483 participants) identified that there was a very low certainty of evidence that RER was not different between the luteal versus follicular phases of the MC at rest (SMD = 0.05, 95% CI = −0.15, 0.25, *P* = 0.63; Fig. 4) or during exercise (SMD = 0.05, 95% CI = −0.13, 0.23, *P* = 0.57; Fig. 5). Analysis of the OC phase from only six studies (*n* = 51 participants) identified that there was a low certainty of evidence, and that RER was not different between the active and inactive OC phase during exercise (SMD = −0.36, 95% CI = −0.76, 0.03, *P* = 0.07).

Ours is the first systematic review and meta-analysis to examine the effect of both MC and OC phases on substrate oxidation at rest and during moderate-intensity continuous exercise. We showed that substrate oxidation at rest and during moderate-intensity continuous exercise was not different between the different phases of the MC. The same was true of substrate oxidation differences between OC users during the active and placebo phases, although we are far less confident in these findings. Overall, the studies included in this analysis provided very low certainty of evidence as assessed by GRADE criteria but reported consistent effects between studies as indicated by relatively low heterogeneity scores.

The relevance of the follicular versus luteal phase is a function of the hormonal effects on substrate oxidation, accepting that MC can be subdivided into at least six subphases (Fig. 1*A*). Yet, data are sparse on how substrate oxidation differs outside the broader binary phase-based definition of the MC (i.e., follicular and luteal). The follicular phase typically has higher estrogen and lower progesterone (Fig. 1). Nonetheless, no MC phasic differences in substrate oxidation were observed, indicating that the magnitude of hormonal fluctuation was insufficient to modulate substrate oxidation at rest or during exercise.

Like the analysis on MC phase, the current meta-analysis revealed no difference between OC phases on substrate oxidation during exercise. One of the most important considerations regarding OC use is the formulation of hormones used in the contraceptive. Ethinyl estradiol, the synthetic form of E2, may bind to estrogen receptors with greater affinity than endogenous estradiol, possibly exerting more potent effects (65). In addition, the progestins in OC act differently compared with endogenous progesterone. First- and second-generation OCs exert differential effects compared with third- and fourth-generation OCs, as the progestins in the former are more androgenic and have been proposed to exert different effects than the latter (66). The two most notable androgenic progestins are levonorgestrel and norethindrone; however, evidence for their androgenicity includes no human data showing any effects on skeletal muscle (66). Nonetheless, it is also important to note that androgenic progestins such as levonorgestrel and norethindrone also have antiandrogenic effects by suppressing serum levels of androgens produced by the adrenal and ovarian systems (66). For example, the adrenally produced androgen marker, dehydroepiandrosterone sulfate and dihydrotestosterone and 3α-androstanediol glucuronide, markers of ovarian androgen production, were similarly and significantly reduced from baseline in those taking levonorgestrel and norethindrone (67). These data (67) indicate that the oral administration of levonorgestrel and norethindrone, likely in combination with ethinyl estradiol, has marked antiandrogenic effects with respect to androgen production. As Alexander et al. (68) point out, when synthetic hormones are blended, it is challenging to distinguish the separate effects of each hormone, similar to the challenges associated with determining the effects of E2 and P4 on the menstrual cycle.

Although hormones in the menstrual and OC cycles could affect substrate oxidation, as seen in cell and rodent studies looking at isolated effects of these hormones, their impact in females is subtle and overridden by more potent factors such as nutritional status, training status, and exercise duration or intensity. Our finding of no phase-based effects on substrate oxidation indicates that there may be no need to control MC phases or OC use, especially if other controls (i.e., nutritional status, exercise intensity, duration, and participant characteristic matching) are in place. Thus, future studies may confidently recruit females independent of phase status within an MC or OC cycle, encouraging greater inclusion of females in substrate oxidation-focused investigations.

## EXERCISE AND SPORTS PERFORMANCE ACROSS THE MC

Although a small number of laboratory-based investigations have shown that ovarian hormones can affect some characteristics of exercise capacity (9), there are fewer studies on the impact of the MC on real-world sporting performance. In reviewing this sparse literature, McNulty et al. (9) concluded that exercise performance might be trivially reduced during the early follicular phase of the MC compared with all other cycle phases. This finding may be meaningful to athletic populations; however, the authors caution that the evidence was of low-to-moderate quality given the design, methodology, and high variability between studies. As such, rather than creating general guidelines, the authors recommend a personalized approach based on each individual’s response to exercise performance across the MC (9). Moreover, exercise performance may not be translatable to “real-world” sports performance. Of the available data, only one out of the 51 (∼1%) strength outcomes and seven out of 80 (∼6%) endurance outcomes studied closely reflect what we would generally recognize as “real-world” sports performance.

Conversely, many studies assessed the effect of MC phase on outcomes such as isometric contractions, single-limb exercises, time-to-exhaustion protocols, or sprint or endurance capacity tests, which do not always reflect sport-based tasks. Finally, most cohorts included in this analysis would not be considered athletes. Just 3 out of the 78 (∼2%) studies used what would be classified as athlete cohorts, defined as Tier 3 and above (see Ref. 69) for definitions and grading of athletes). The lack of athletic cohorts may result from many elite athletes not naturally cycling. Recently, Oxfeldt et al. (70) reported that 57% of elite female athletes, Tier 3 and above (69), in Denmark use hormonal contraceptives, with 74% using combined (estrogen and progesterone) and 26% using progestin only. Interestingly, 60% of OC users reported having manipulated their MC by continuous OC use, i.e., no placebo phase. As such, McNulty et al.’s (9) findings that performance might be trivially reduced during the early follicular phase may not be relevant nor applicable to athletic populations. Instead, a personalized approach to assessing individual exercise responses across the MC is recommended in eumenorrheic athletes.

The effects of the MC on real-world sport performance outcomes produces mixed findings. According to Greenhall et al. (71), more than half (57%) of recreational marathon runners self-reported personal best marathon times during the luteal (defined by the authors as the last 14–15 days of participants self-reported MCs) rather than follicular (defined by the authors as the first 14–15 days of participants self-reported MCs) phase of their cycles. This trial, however, did not confirm ovulation and assumed a MC length of 28–30 days, along with equal lengths of follicular and luteal phases across all study participants (71). Failure to characterize the MC phases more carefully, including ovulation testing (10), is a limitation of this trial, and thus the true phases cannot be verified. Another study in track and field athletes found that 100 m and 200 m running performance was significantly faster during the luteal (days 19–23 of the cycle) phase; however, no difference between menstrual phases was seen in the 2,000 m event (72). In this trial, the phases were verified using blood-based hormonal analyses, with the luteal phase characterized by higher P4 levels than during the follicular phase but not higher E2 levels, suggesting a supporting effect of P4 or a synergistic effect of P4 and E2 on performance on shorter distance events.

In addition, when assessing menstrual cycle effects on exercise and sports performance, it is important to acknowledge that performance changes may not primarily stem from variations in hormonal concentrations but rather from the symptomology associated with the menstrual cycle. Athletes often employ OC to help mitigate the effects of MC symptoms on performance (70). An interesting finding from a well-controlled study (73) identified that, unlike naturally cycling women, those on OC did not experience phase differences in psychological and physical well-being or exercise performance. In contrast, in eumenorrheic women, it was found that counter-movement jump performance and Wingate average power were lower at the end and start of the MC, respectively, whereas no significant difference in strength (handgrip and isometric elbow flexor strength) was observed across the phases. Notably, the authors did not observe any significant correlation between variations in sex hormone levels and exercise performance. However, they did observe performance variations to be correlated with psychological factors such as motivation, affect, perception of their performance, and indicators of well-being such as pain (73). Eumenorrheic women in this study reported higher levels of physical pain and lower pleasure; the participant’s perception of their performance level was lower in the EF. Similarly, a study of Australian athletes training for the Olympic and Paralympic Games revealed that two-thirds of athletes perceived that their MC affected performance (74). Approximately 80% of these athletes identified a specific phase of the MC that they thought was optimal for their performance, with the majority (54%) describing this as the time frame “just after their period.” In another study, participants reported that their MC either negatively (50%) or positively (6%) affected training, particularly just before (1–2 days) and during menstruation (73). Furthermore, 56% of athletes self-reported the perception that match-day performance was negatively affected by their MC, with the most frequently reported aspects of performance impacted being energy levels, fatigue, and endurance capacity (73).

The variability seen in the impact of fluctuations in E2 and P4 associated with the MC on field-based tasks may be explained by various factors. These factors include differences in the chosen performance task, the caliber of athletes, and, most importantly, the methodological design to achieve desired hormonal differences. Unfortunately, many investigations have failed to properly assess the actual MC phase or status of the study participants, including confirming ovulation. Field-based research is particularly challenging as there are typically larger performance measurement errors compared with laboratory environments due to the influence of extraneous variables such as environmental conditions or the overlay of skill and technique. Accordingly, the ability to detect small changes in performance due to hormonal fluctuations becomes more challenging. Despite these difficulties, it is important and necessary to determine the field-based implications of any laboratory findings before attempting to change the real-world practices of athletes.

In addition, while the aforementioned data suggest that the MC and associated symptoms can affect an athlete’s perception of performance, some limitations should be considered. First, there is currently no valid or reliable way of assessing menstrual symptoms in elite female athletes, with issues around construct validity and confirmation bias inherent in most measures used to determine the impact of the MC on performance. Furthermore, most previous work (74) has used a cross-sectional design, and there are few prospective studies (73) implementing performance tests alongside verified MC phase occurrence (10). In addition, as mentioned previously, there is substantial intraindividual variability regarding the impact of menstrual symptoms on training and performance. Lastly, we have yet to determine if the symptoms reported are exclusively MC-related or are a combination of MC plus other factors (e.g., lifestyle, injury, illness, recovery, nutrition). Therefore, we suggest that individualized approaches are taken when managing menstrual symptoms (after they have been verified as MC symptoms) to optimize performance in athletes.

## EFFECTS OF MC PHASE ON SKELETAL MUSCLE ADAPTATIONS WITH RESISTANCE TRAINING

Skeletal muscle can undergo remodeling with resistance exercise training, resulting in hypertrophy; see Ref. 68 for a review. We have previously documented the weak evidence supporting particular MC phases as being associated with greater resistance training-induced hypertrophy (13), whereas others have concluded that phase-based training confers some advantages (11, 75). The rationale behind this hypothesis is often that estrogen, the “dominant” hormone in the follicular phase, allows for greater anabolism; however, it has been argued that limited human evidence supports this notion.

Preclinical models suggest that E2 (follicular) or E2 and P4 (luteal) predominance could ostensibly affect resistance training outcomes. Ovariectomized (OVX) rodent models are often used to illustrate the proof-of-concept for hormone effects on skeletal muscle. However, while OVX models may yield mechanistic insight into estrogen-mediated muscle repair and regeneration, it is important to consider the inability to translate OVX-derived findings (except for females undergoing a radical hysterectomy) to human models, and this is especially so in the context of understanding the effect of MC phase on muscle repair and regeneration. Briefly, OVX animals are models of complete sex hormone deficiency compared with animals with cyclical hormones (76), in contrast to a wide range of concentrations and durations of E2 and P4 in female exercise physiology research (Fig. 1*F*). In humans, a recent study showed regional gains in muscle cross-sectional area to be greater with resistance training in postmenopausal women on transdermal estrogen than those using a placebo (77); however, this result was inconsistent between the sites measured. The greater single-site growth was also not associated with any functional (strength) advantage, so the benefit of estrogen-induced muscle growth in humans is somewhat questionable. We briefly review some potential mechanisms and include a critical commentary on the possibility that hormones such as E2 and P4 influence hypertrophic outcomes in females undertaking resistance training.

Muscle-specific stem cells or satellite cells play a role in hypertrophy and have been shown to be involved in the hypertrophic process (78). Evidence suggests that E2 may mediate satellite cell activation through estrogen receptor alpha (ERα) and beta (ERβ), ligand-activated transcription factors. When E2 binds to ERα/β, the complex translocates into the nucleus, where it binds to estrogen-responsive elements in DNA, activating the transcription of myogenic-promoting factors and genes related to muscle regeneration (79, 80). Work in rodents (81) and humans (76) has shown beneficial estrogen-related effects on muscle damage, regeneration, and repair through cell membrane stabilization and influence on SC activation and proliferation (76, 82). However, the relevance of these observations for hypertrophy and muscle remodeling is not entirely clear. A recent review (83) examining the role of estrogen in satellite cells found that the menopausal transition, which, in the authors’ view, was mimicked by the ovariectomized rodent model, was associated with a loss of satellite cells. However, these authors (83) concluded, “…hormonal fluctuations during the menstrual cycle or a changed hormonal milieu due to the use of hormonal contraceptives, [that] the evidence is still limited and results inconclusive in regard to the role of SCs [satellite cells].”

Haines et al. (80) sought to investigate whether differences in E2 levels across the MC could affect markers of muscle regeneration following eccentric exercise in young, healthy eumenorrheic females. The authors compared serum and muscle estradiol concentrations, ERα protein content, and the binding of ERα to DNA postexercise in the midfollicular and midluteal phases (80). The authors also examined the expression of certain genes related to muscle regeneration, including ERα and MyoD mRNA (80). While serum E2 levels were significantly lower in the MF than in the ML phase, there was no difference in muscle concentrations of E2. However, we question the accuracy of the method used by these authors to determine muscle E2 concentrations. They used ELISA manufactured to analyze serum hormone concentrations, which was not validated for tissue homogenate, on a muscle homogenate. In our view, this practice would not have accurately determined intramuscular hormone concentrations. The usual method of accurate measurement is to extract hormones from tissue and analyze them using mass spectrometry analysis; see Ref. 84. Haines et al. (80) additionally found that ERα mRNA and ERα protein content expression was slightly, but statistically, greater in the MF phase; however, the binding of ERα to DNA and Myo-D expression did not differ between MC phases. Haines and colleagues (80) concluded that ERα activation in response to eccentric exercise is independent of serum E2 levels, supporting the conclusion that muscle regeneration and repair do not differ due to cyclic changes in E2.

Few human studies have sought to investigate the role of MC phase-directed training on muscular strength and hypertrophy. Nevertheless, several studies (85–87) are often used to give ostensible support for prescribing MC phase-based training to enhance skeletal muscle adaptations (11) despite findings from other studies of a lack of benefit to phase-based training (88). In all interventions (85–87), researchers attempted to manipulate resistance exercise training volume around the MC to take advantage of the hormonal environments associated with each phase. The aim of controlling exercise volume was to maximize strength and hypertrophic gain (85–87) with the thesis that E2 is anabolic and P4 is catabolic (or at least antianabolic) and so antagonizes the anabolic effect of E2 and suppresses anabolism during the luteal phase of the MC. These studies (85–87) reported that females that trained with a higher volume of resistance exercise training in the follicular phase of their MC (often defined as the 2 wk beginning at the onset of menses) observed greater gains in muscle strength and hypertrophy than those that trained with higher volumes in their luteal phase (85, 87). It was reported that those who trained consistently throughout their cycle (86, 87) had lesser gains in muscle mass than those training with greater volume in their follicular phase, which is a conclusion that is particularly difficult to reconcile, given the agreement around the influence of resistance training volume on strength and hypertrophy outcomes (89). We have previously documented the lack of methodological rigor when assessing and tracking MC phase in these studies, which limits confidence in their findings (13, 90). A recent review recommended that women periodize their training around their MC (11); however, we find these recommendations to be without a good basis due to contrary findings (88) and a lack of high-quality mechanistic and chronic training studies to suggest that this practice results in a benefit (13). We also argue that these recommendations lack practicality, whereas adjusting a trainee’s training based on their menstrual cycle phase would require a switch in training styles on a MC phase length basis, which could be decidedly asymmetrical (a prolonged follicular vs. a shorter luteal phase or vice versa) between subsequent MCs. A more pragmatic approach is for the individual to train consistently throughout their MC to maximize the potential benefits of their practice while adjusting according to their self-reported fatigue levels and readiness to train. Such an approach reflects the fundamental basis of programming training sessions regardless of sex.

We acknowledge that different generations of OCs may have different effects. For example, third- and fourth-generation OCs have shown more favorable side-effect profiles due to their less androgenic profile (91). Current literature investigating the effect of OC use on acute strength measures and adaptation to resistance exercise training is sparse and needs studies employing greater control and gold-standard levels of practice (10) to understand the true effect of OCs on strength and chronic resistance training outcomes (75).

A limited number of studies have been conducted on muscle protein synthesis (MPS) measurements with changes in female hormonal status. Appreciating that MPS is part of the process of resistance training-induced muscle hypertrophy (78) and not always indicative of hypertrophy itself (92), there are learnings from this work that we propose are instructive on a mechanistic level in humans. Smith et al. (93) administered E2 to postmenopausal females for 14 days, raising their endogenous E2 levels to those seen in premenopausal females during their mid- to late-follicular phases, and showed no change in MPS or markers of muscle proteolysis. Park et al. (94) used acute E2 administration and observed increases in forkhead box O3 (FOXO3) activation (phosphorylation) and muscle-specific ring finger protein1 (MuRF1) protein content in late (≥10 yr) but decreased in early (≤6 yr) postmenopausal females. The acute (94) versus longer-term (93) protocols may be a critical difference.

**Figure 6.**
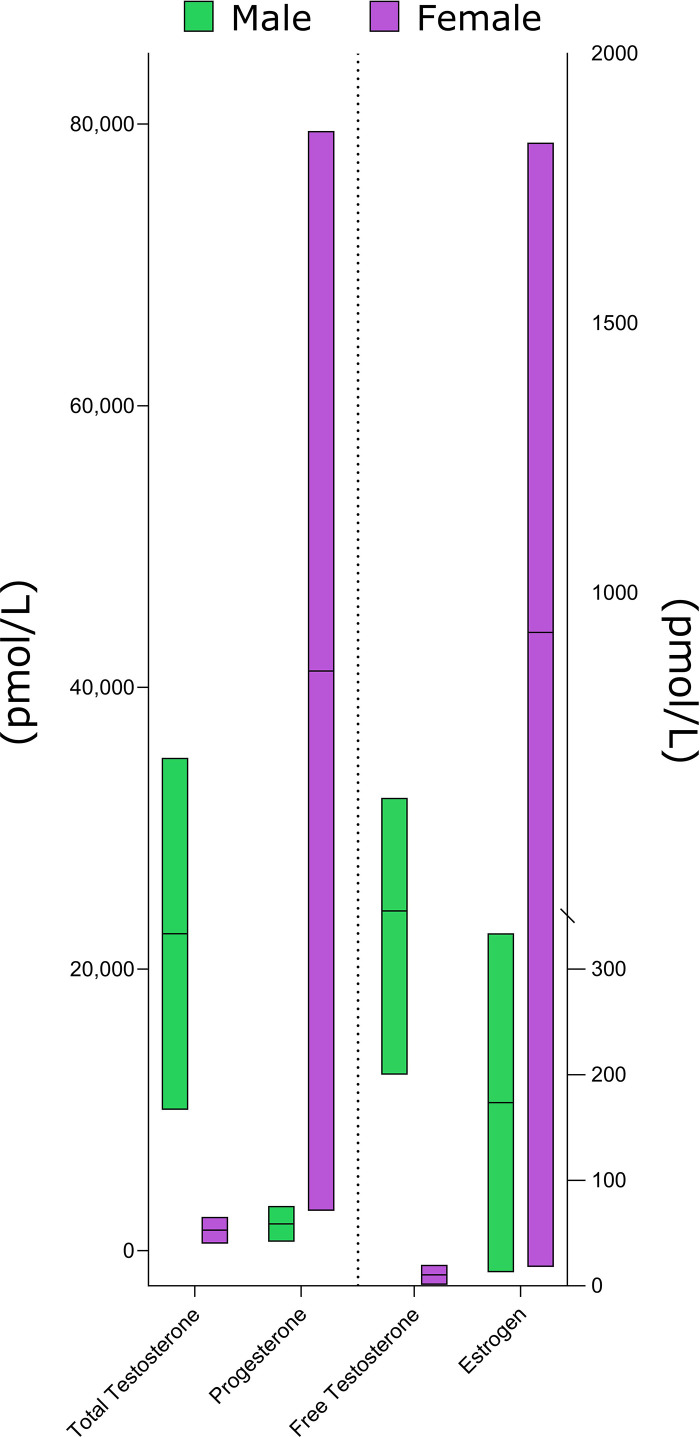
A schematic depicting normal hormone concentration ranges in males and females with concentrations taken from Refs. 6–8.

As opposed to E2, vaginal administration of micronized P4 (resulting in P4 levels equivalent to those seen in the midluteal phase of premenopausal women) increased MPS comparable to that seen with transdermal administration of testosterone (93). Such an observation concurs with the androgenic nature of P4 versus E2 (66) but contrasts with statements that the follicular is a more anabolic phase due to higher relative levels of E2 and that greater resistance training volumes would be beneficial to enhance hypertrophy (85–87). We also note that given the known androgenic profile of testosterone, and despite substantial sex-based differences in total and free testosterone concentrations (Fig. 6), when females are compared with males, they exhibit very similar relative hypertrophy in response to resistance training (95). As Smith and Mittendorfer (96) have pointed out, the sharp divergence in muscle mass occurs at puberty, but after ∼18–20 yr of age, males and females gain and lose, on a relative basis, similar amounts of muscle mass when exposed to loading or unloading, respectively. There has been speculation, based on static snapshots of markers of proteolysis (97) and enzymatic appearance in blood after “damaging” (eccentrically biased) exercise protocols (98) that estrogen could alter protein breakdown; however, there are no reports that we are aware of where proteolysis has been directly measured. We note that a meta-analysis of hormone replacement therapy in postmenopausal women showed no impact on lean mass (99).

## VASCULAR OUTCOMES AND MC PHYSIOLOGY

An illustrative example of how estrogen affects vascular function can be seen in female aging particularly around menopause. The cardiovascular system exhibits age-related deterioration of vessel function and arterial stiffness that is more prominent in males until menopause, where dysfunction accelerates in females. For example, research has identified that endothelial function decreases across stages of perimenopause to early and late postmenopause stages (100). Impairments in cardiovascular function with menopause also are evident in studies examining arterial stiffness (101), blood pressure (102), and cardiac function (103). These observations indicate a potential for a “female advantage” or protective influence of the MC, specifically E2, in offering cardiovascular protection, potentially through antioxidant properties and effects on the nitric oxide synthase pathway (104, 105). A critical piece of evidence in this hypothesis is the obligatory role E2 plays in inducing improvements in endothelial function with aerobic endurance exercise training in postmenopausal females (106). Here, we examine the subtle-to-absent influence of the MC on cardiovascular outcomes; the majority of research has been conducted at rest, though some evidence from exercise studies is also reported.

The MC may have a subtle influence on local carotid artery stiffness (107), but there is minimal evidence to suggest an effect on the peripheral vascular stiffness (107–110) or structurally on carotid intima-media thickness (107). While several studies have not observed differences in carotid artery β-stiffness across the MC (108, 110), one study showed a decrease in β-stiffness during ovulation and an elevation in distensibility during the menses to ovulatory period compared with luteal phases (107). Similarly, recent research showed an increase in shear-mediated dilation of the carotid artery during the late-follicular compared with the early-follicular phase and that dilation was positively associated with serum E2 levels (111). While research examining the intersections of the MC, exercise, and arterial stiffness is limited, one study by Okamoto et al. (112) showed an augmented arterial stiffening response acutely following a bout of resistance exercise in the follicular, but not in the luteal phase of the MC.

In the case of resting hemodynamics, there is no evidence of an impact of the MC on aortic or brachial blood pressure (109–111, 113, 114) and little (∼2 beats/min difference) to no influence on resting heart rate (HR) (109, 111, 113–115). Elevated resting HR during the ovulatory or luteal phases of the MC may be attributed to elevated blood volume or body temperature, though confirmatory research is needed (116). There was no impact of MC phase on exercise HR during two moderate-intensity continuous exercise tests (117), but an apparent increase in HR during the luteal phase at 65% V̇o_2peak_ during a continuous exercise test (118). There may be an effect on reducing HR variability and thus increasing sympathetic activity during the luteal versus follicular phase in trained females (119); however, these results are mixed, potentially due to a longer assessment duration than the former two studies (115). There are discrepant findings on the influence of the MC on left ventricular function. One study showed a shorter left ventricular ejection time in the midluteal compared with the follicular phase, though the authors question the clinical impact of this finding (113). Similarly, one study observed decreased myocardial performance and decreased isovolumic contraction and relaxation time (though not ejection time) in the luteal, compared with menstrual phase (120); however, these findings have not been corroborated in other studies (114) and further research is needed examining cardiac function at rest and during exercise.

Vascular function has been well studied at rest, showing no influence of the MC on smooth muscle function but a potential subtle and variable effect on endothelial function, only present in the macrovasculature (121). Early research on endothelial function showed a robust increase in endothelial and smooth muscle function in the microvasculature in the late follicular and midluteal phases. While these early findings have been corroborated, with a smaller magnitude of change, several studies following gold-standard guidelines have not observed this effect (122, 123). Furthermore, a recent meta-analysis showed no impact of the MC on macro- or microvascular smooth muscle function and a small effect on macro-, but not microvascular endothelial function (121); however, this may be attributed to methodological differences in adherence to guidelines. The decision to “control for” the MC has been debated (124, 125), recently culminating in a point-counterpoint series (126) concluding that the MC has a minor effect on endothelial function and should only be considered when a priori hypothesis would make the issue relevant and where resources and study design permits. Adding to the complexity of studying the MC, there is considerable variability in the MC (Figs. 1*F*, 2, and 3), making it challenging and begging the question of whether controlling for MC phase is pragmatic. For example, research by Liu et al. (127) identified substantial intraindividual variability in the changes across early- to late-follicular phases across two MCs. While the effect of the MC on smooth muscle and endothelial function is relatively well understood, the interaction with exercise is absent from the literature and warrants future investigation.

## THE EFFECTS OF ORAL CONTRACEPTIVE USE ON CARDIOVASCULAR ADAPTATIONS AND EXERCISE PERFORMANCE

Research on the influence of OC on the cardiovascular system is limited and largely based on research examining OC users compared to nonusers (i.e., naturally cycling females) at rest. A recent review by our group (128) identified that OCs did not influence micro- and macrovascular smooth muscle function or arterial stiffness but may impact endothelial function. Specifically, some studies have shown impairments in endothelial function associated with second generation OC use (91), but either no change or improvements with third and fourth generation OC use (129, 130). There is evidence that BP is elevated in the active OC phase (109), though this has not been replicated (110). However, there is no difference in muscle sympathetic nerve activity between OC users and nonusers (131) and across OC phases (132).

Research on cardiovascular responses to exercise is scarce; however, most studies have reported minimal to no alterations in cardiovascular outcomes with OC use or between OC phases. For example, cardiac vagal withdrawal at the onset of exercise, heart rate variability, or other hemodynamic measures were not different across OC users versus nonusers or hormonal phases (133, 134). However, heat stress (i.e., increased core body temperature) during exercise may be increased in the active versus placebo OC phase (135), and skin blood flow response during heating in OC users compared with nonusers (136), though the cardiovascular implications of this small increase in heat stress are unknown. Further research is needed to examine the influences of generations of OC use on cardiovascular responses to exercise.

In a recent meta-analysis by Elliott-Sale et al. (137), these authors concluded that, on average, OC users might have trivial impairments in acute measures of exercise performance when compared with their non-OC counterparts. Notably, the authors highlight the nuances that need to be considered when applying these findings practically (137). Exercise performance can be partitioned into strength and aerobic performance, often quantified through key performance indicators such as maximal force output and V̇o_2peak_. Given the different metabolic pathways used in aerobic versus strength exercise, OC use may have differential effects, depending on the outcome.

## CONCLUSIONS

We based our findings in this review on data derived from humans. We acknowledge that some areas have less evidence than others and, importantly, the methodological quality of trials in these areas is frequently poor. Nonetheless, we propose that much of the previous literature on the ostensible effects of MC impacts on human physiology is a blend of observations from OVX animal and cell-based models to substantiate weaker and fewer observations in humans. Such an approach is also sometimes clouded by including studies with poor methodological approaches and a lack of verification of hormonal status. This approach has resulted, in some cases, in a mischaracterization of the effects that sex steroids have on performance, metabolism, vascular effects, and resistance training-induced hypertrophy. The often-cited axiom reminds us that absence of evidence is not evidence of absence. Nevertheless, based on current evidence and critical review, the differences between MC phases, if present, are small, at least based on the outcomes we reviewed. Other areas, for example, substrate oxidation, where a large body of data exist, and our meta-analysis clearly show no impact of MC phase on the balance of fat and carbohydrate oxidation at rest or during exercise.

We acknowledge that conclusions around the MC and the need for control are debated in some research fields (124, 125). We strongly urge that females receive greater attention in exercise physiology research, as their inclusion in physiology studies is woefully low (1–5). While we find scant evidence to recommend that the MC needs to be controlled for or that its presence would be a reason not to include females alongside males in certain studies, we suggest that if the a priori hypothesis demands it, perhaps in newer areas of research or where there is a compelling reason to do so, we encourage those working in this area to use rigorous methodologies to monitor MC status. Without such rigor, any potential differences, as small as they may be, are unlikely to be revealed. Similarly, if females are being compared with males regarding their responses to aspects of exercise training, the study should be appropriately powered to detect differences in the outcomes of interest if one is expected. These practices will deepen our understanding of this area, including best practices outlined here and detailed previously (10). Overall, the consensus of this review suggests that the impact of MC and OC use on various physiological outcomes is small or, in many cases, nonexistent. We strongly encourage the inclusion of females in more rigorous physiological research.

## SUPPLEMENTAL DATA

10.6084/m9.figshare.23279927 Supplemental Figs. S1 and S2: doi.org/10.6084/m9.figshare.23279927.

## GRANTS

This work is supported by Ontario Women's Health Scholar (to J.S.W.), Canada Research Chairs (Chaires de recherche du Canada) (501100001804; to S.M.P.), Gouvernement du Canada | Canadian Institutes of Health Research (IRSC) (501100000024; to M.A.T. and S.M.P.), Gouvernement du Canada | Natural Sciences and Engineering Research Council of Canada (NSERC) (501100000038; to A.C.D., M.J.M., G.P., M.A.T., and S.M.P.).

## DISCLOSURES

S.M.P. reports personal fees from Nestle Health Sciences and nonfinancial support from Enhanced Recovery outside the submitted work. S.M.P. has patents licensed to Exerkine but reports no financial gains from patents or related work. M.A.T. is President and CEO of Exerkine Corporation who develops postexercise recovery drinks for athletes but none that are sex-specific. None of the other authors has any conflicts of interest, financial or otherwise, to disclose.

## AUTHOR CONTRIBUTIONS

A.C.D., M.W., J.S.W., and S.M.P. conceived and designed research; A.C.D. and S.M.P. prepared figures; A.C.D., M.W., J.S.W., L.M.C-S., D.G.M., A.K.A.M., K.J.E-S., L.M.B., and S.M.P. drafted manuscript; A.C.D., M.W., J.S.W., L.M.C-S., D.G.M., A.K.A.M., K.J.E-S., L.M.B., G.P., M.J.M., M.A.T., and S.M.P. edited and revised manuscript; A.C.D., M.W., J.S.W., L.M.C-S., D.G.M., A.K.A.M., K.J.E-S., L.M.B., G.P., M.J.M., M.A.T., and S.M.P. approved final version of manuscript.
